# GEMIN4, a potential therapeutic targets for patients with basal-like subtype breast cancer

**DOI:** 10.1186/s12905-023-02547-1

**Published:** 2023-07-28

**Authors:** Liang Wu, Yue Zhang, Chunlei Zheng, Fuqiang Zhao, Yan Lin

**Affiliations:** 1 Department of Emergency Surgery, The Second Affiliated Hospital of Qiqihar Medical University, Heilongjiang Province, Qiqihar, People’s Republic of China; 2 Department of International Education School, Qiqihar Medical University, Heilongjiang Province, Qiqihar, People’s Republic of China; 3 Department of Surgical Oncology, The Second Affiliated Hospital of Qiqihar Medical University, Heilongjiang Province, Qiqihar, People’s Republic of China; 4 Department of School of Basic Medicine, Qiqihar Medical University, Heilongjiang Province, Qiqihar, People’s Republic of China

**Keywords:** Basal-like breast cancer, GEMIN4, Weighted gene co-expression network analysis, Prognosis

## Abstract

**Background:**

Basal-like breast cancer (BLBC) takes up about 10–20% of all breast cancer(BC), what’s more, BLBC has the lowest survival rate among all BC subtypes because of lacks of efficient treatment methods. We aimed to explore the molecules that can be used as diagnostic maker for BLBC at early stage and provide optimized treatment strategies for BLBC patients in this study.

**Methods:**

Apply weighted gene co-expression network analysis (WGCNA) to identify gene modules related to BLBC;The functional enrichment of candidate genes related to BLBC in the red module of Go data package and KEGG analysis;Overlapping cross analysis of URGs and WGCNA to identify candidate genes in each BC subtype;Divide BCBL patients into high-risk and low-risk groups, and analyze the two groups of overall survival (OS) and relapse free survival (RFS);Screening of GEMIN4 dependent cell lines; QRT PCR was used to verify the expression of GEMIN4 transfected with siRNA; CCK8 was used to determine the effect of GEMIN4 on cell viability; Positive cell count detected by BrdU staining;GO and KEGG enrichment analysis of GEMIN4.

**Results:**

The "red module" has the highest correlation with BLBC, with 913 promising candidate genes identified from the red module;913 red module candidate genes related to BLBC participated in multiple GO terms, and KEGG enrichment analysis results mainly enriched in estrogen signaling pathways and pathways in cancer;There are 386 overlapping candidate genes among the 913 "red module" genes identified by 1893 common URG and WGCNA;In BLBC patients, 9 highly expressed genes are associated with OS. Five highly expressed genes are associated with RFS. Kaplan Meier survival analysis suggests that high GEMIN4 expression levels are associated with poor prognosis in BLBC patients;The GEMIN4 gene dependency score in HCC1143 and CAL120 cell lines is negative and low; Si-GEMIN4-1 can significantly reduce the mRNA expression of GEMIN4; Si-GEMIN4 can inhibit cell viability; Si-GEMIN4 can reduce the number of positive cells;GO enrichment analysis showed that GEMIN4 is associated with DNA metabolism processes and adenylate binding; KEGG pathway enrichment analysis shows that GEMIN4 is related to ribosome biogenesis in eukaryotes.

**Conclusion:**

We hypothesized that *GEMIN4* may be the potential target for the treatment of BLBC.

**Supplementary Information:**

The online version contains supplementary material available at 10.1186/s12905-023-02547-1.

## Introduction

Breast cancer(BC) is the commonest type of carcinoma in women and it has a high mortality rate[1]. According to the Global cancer statistics 2018, the global incidence and deaths of BC are estimated to be 2 million and 0.6 million in 2018, respectively [2]. During the past three decades, the morbidity, mortality, as well as disability-adjusted life-year (DALY) of BC kept increasing worldwide [3]. The major well-defined risk factors for BC could be clustered into two aspects, individual and reproductive. The individual factors are including sex, age and hereditary, and the reproductive factors are contain early menophaaria, acocia, menopause, and delay first-time birth [1]. Therefore, early diagnosis and treatment of BC, improvement of disease prognosis and optimization of treatment decisions have become an urgent public health issue.

Different systems of classification have been implemented in the clinic to improve prognosis and optimize treatment decision-making of BC. Based on the gene expression patterns, BC tumors are currently classified into six subtypes: normal-like, Her2-enriched, claudin-low, basal-like, luminal A as well as luminal B [4]. Basal-like breast cancer (BLBC) is the highest-grade invasive BC. The BLBC presented low expressions of progesterone receptor (PR), estrogen receptor (ER), as well as human epidermal growth factor 2 receptor (HER2), thus also be recognized as a subtype of triple-negative breast cancer (TNBC) which takes up about 10–20% of all BC [5, 6]. What’s more, BLBC has the lowest survival rate among all BC subtypes because of lacks of effective and established treatments [7, 8]. Present first-line clinical treatment for BLBC was chemotherapy, the drug and surgery therapies were not appropriate to the patients with BLBC due to out of definite drug targets as well as surgical conditions [9]. Therefore, exploring biomarkers and patients driver genes shown vary important and urgent for disease monitoring and treatment of BLBC.

In this study, we compared the differentially expressed genes (DEGs) between BLBC and other five subtypes and gene modules most related to BLBC identified by weighted gene co-expression network analysis (WGCNA). The common genes were then intersected with the potential target genes involving the relapse free survival(RFS) and overall survival(OS) of BLBC to explore potential biomarkers that affect the prognosis of BLBC and the molecular mechanism of its occurrence and development.

## Materials and methods

### Data source and preparation

**Fig. 1:**
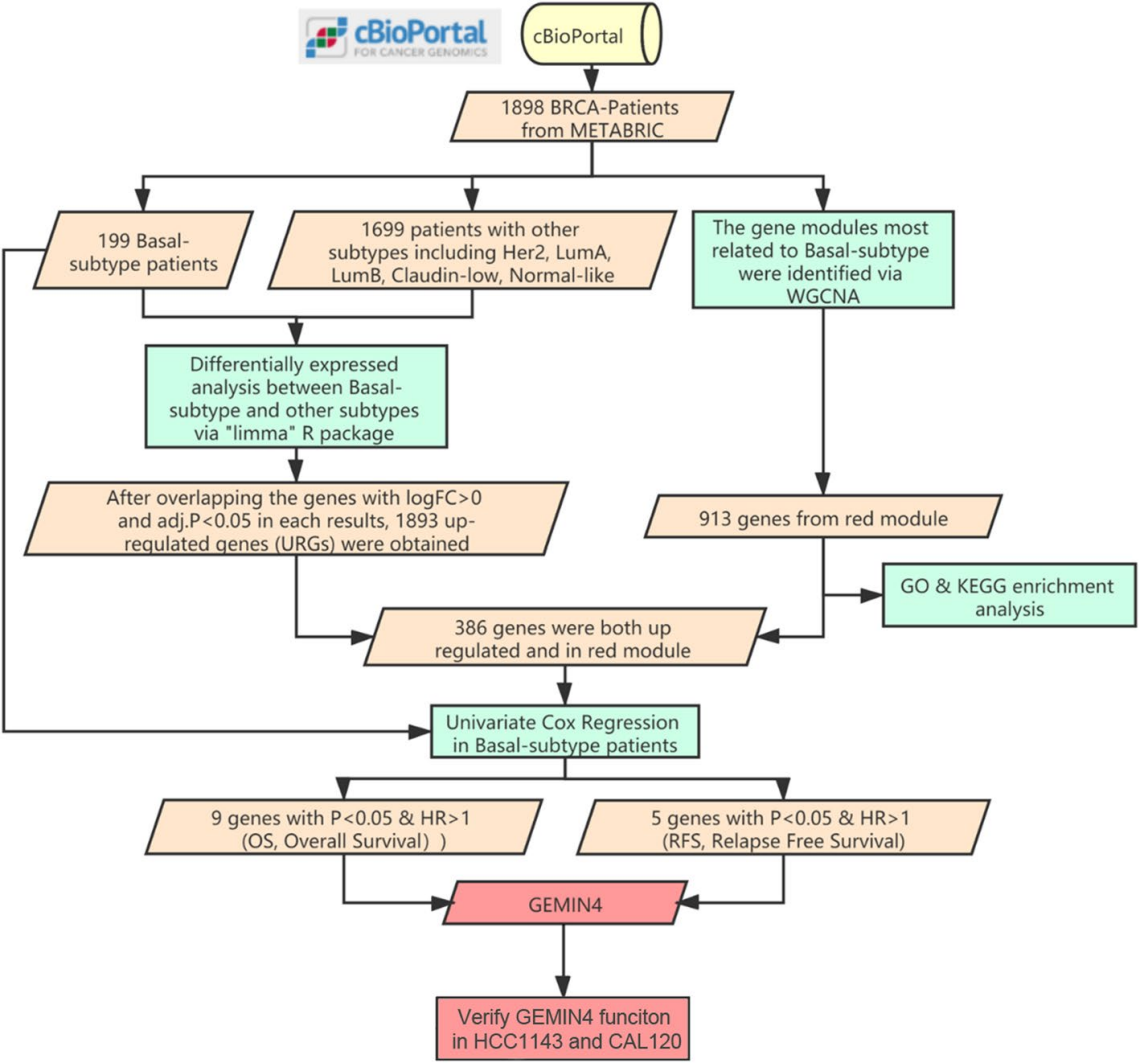
The workflow of this study

The Molecular Taxonomy of Breast Cancer International Consortium (METABRIC) dataset were downloaded from cBioportal (http://www.cbioportal.org/) [10]. Specifically, select the Breast item on the front page of the cbioportal database, The dataset in Breast Cancer (METABRIC, Nature 2012 & Nat Commun 2016) was selected from the invasive breast carcinoma column for download. 1,898 BC patients (199 BCBL and 1,699 other subtypes, including normal-like, Her2-enriched, claudin-low, luminal A as well as luminal B) with expression profiles, survival data as well as clinical features were enrolled in current research. The human protein-coding genes were annotated according to the GENECODE (https://www.gencodegenes.org/) and included in the subsequent analysis. Supplemental Table 1 shows the detail clinical characteristics of BCBL patients and Fig. 1 shows the workflow of this research.

### *Identification of gene modules related to BLBC *via* WGCNA*

WGCNA analysis was applied to clusters genes for each of the BC subtype based on their gene expression matrix. The appropriate soft-threshold power for network construction was provided by calculating the scale-free topology fit index for several powers using “WGCNA” R package [11]. In the BC subtypes, the interactions between genes were calculated through the values of Gene significance (GS), and the module membership (MM) indicated module eigengenes of gene expression profiles. Totally 913 candidate genes from the ‘red module’ with the highest correlation coefficients were selected for subsequent analysis. A total of 913 candidate genes with the highest correlation coefficients were selected from the "red module" for subsequent analysis.

### Functional enrichment analysis of the candidate genes selected from ‘red module’ associated with BCBL

The Web-based gene set analysis toolkit (WebGestalt) (http://www.webgestalt.org/) [12] online tool was used for GO (including molecular function (MF), cellular component (CC), as well as a biological process (BP) and KEGG pathway enrichment analysis of the candidate genes related to BLBC in red module. The "over-representation analysis" (ORA) was selected as the ‘Method of Interest’. The results were visualized by "ggplot2" and "ggpubr" R package.

### Identification of DEGs between BLBC and other BC subtypes

DEGs between BLBC and the each of the remaining subtypes were recognized separately by the “limma” R package. And the genes with log2 value fold change (FC) > 0 as well as *p* value < 0.05 after adjusting for FDR were considered as up-regulated genes (URGs). We then intersected the overlapped URGs from each result and identified 1,893 URGs in BLBC compared with other BC subtypes. These URGs were further compared with the 913 genes in ‘red module’ identified by WGCNA. Finally, 386 common candidate genes were identified for BLBC.

### Construction and verification of prognostic value of the 386 candidate genes associated with BLBC

The prognostic significance of these candidate genes was evaluated by univariate Cox regression which performed by the “survival” R package. Based on the median expression value of the 386 candidate genes, BCBL patients were separated into low-risk as well as high-risk groups and the difference in OS and RFS between the two groups were investigated. Genes with hazard ratio (HR) > 1 and *p* value < 0.05 were considered to be a risk factor for BLBC and were selected for follow-up study.

### Biological function analysis of GEMIN4 in BLBC cell lines

Highly expressed *GEMIN4* was appeared to be related to the poor OS and RFS of BLBC. The average expression of *GEMIN4* in BLBC cell lines were downloaded from the Depmap database (https://depmap.org/portal/) and compared with the cell line CRISPR, RNA interference (RNAi) screening data to analyze the impact of *GEMIN4* on the growth ability of BLBC cell lines.

### Molecular mechanism of GEMIN4 in the progression of BLBC

According to the median expression of *GEMIN4* in 199 BLBC patients, the cases were separated into upregulated as well as downregulated groups for Bayesian analysis by using “limma” R package. Bayesian t values of each DEGs (*P* value < 0.05) were obtained for GO and KEGG signal pathway analysis.

### Cell culture

BLBC cell lines including HCC1143 and CAL120 were cultivated in DMEM medium (C11995500CP) with 10% Fetal bovine serum (FBS) (10,091,148) and 100U/mL Pen/Strp (15,070,063). The reagents and media are from Gibco, USA. All cells were fertilized in a 37℃ incubator with 5% CO_2_. The cells were transfected with siRNA when the cell confluence reached about 80%, after 48 h of transfected, the cells were collected for analysis.

### GEMINR small interfering RNA (siRNA) transient transfection

The *GEMIN4* siRNA (si-*GEMIN4*) and negative controls (si-NC) were from GenePharma (Shanghai, China). The transfection of siRNAs with Lipofectamine 30,000 (L3000001, Invitrogen, USA) followed the instruction by the manufacturer, the brief steps were as follows: when the cell confluence reached about 80%, mixed 20uL si-GEMIN4 or si-NC with 5 uL Lipo3000 and standing for 20 min, then added the mixture into the culture medium. After 48 h, drop the culture medium and washed the cells with cold 1 × PBS three times, then collected the cells for the next analysis.

### RT-qRCR

RNAiso Plus reagent (9108, Takara, Japan) was used to extract the total RNAs, and reverse transcribed to cDNA through PrimeScript 1st strand cDNA Synthesis Kit (6110A, Takara, Japan) following the instruction by the manufacturer. Then, the RT-qPCR was used by TB Green Advantage qPCR premixes (639,676, Takara, Japan). The RT-qPCR was performed as follows: 95 ℃ for 15 s, 60 ℃ for 20 s, and 72 ℃ for 15 s, then repeated the cycle for 35 cycles. The threshold cycles (Cts) of each sample were detected by Bio-rad CFX Opus 384 system and each sample was loaded and detected at least three times. The Cts of samples were normalized to the GAPDH Cts with 2^–∆∆Ct^ methods.

### MTT assay for detecting cell proliferation

Cells were counted after trypsin digestion, seeded into 96-well plates with 3,000 cells per well and 6 parallel holes in each group, and cultured in a 37℃ incubator with 5% CO_2_ when BLBC cells were overgrown with the highest and the lowest expression of GEMIN4. The above transfection technique was used to transfect siRNA.

The cells were planted in 96 wells and transfected followed the manufacturer's protocols. After 48 h of transfection, the medium was changed to a 100 µL new culture medium and 20 µL MTT (M1020, Solarbio, China) reagent was added to the new culture medium, then fertilized at 37 °C with 5% CO_2_ for 4 h. Next, added 110 µL of Formazan solution to each well and detected the optical density (OD) at 490 nm.

### Cell proliferation detection

The cells were washed three times by 1 × PBS and fixed with cold 4% PFA for 10 min, then penetrated cells with 0.5% Triton-X100 in 1 × PBS for half an hour. The cells were fertilized by BrdU solutions (B8010, Solarbio, China) for 10 min, and calculated positive staining cells in the fluorescent microscope.

### Statistical Analysis

All experiments were repeated at least five times, with data expressed as mean ± standard deviation (SD). Use the double tailed Student t-test or Mann Whitney test to analyze statistical significance. Evaluate statistical significance using logarithmic rank test. A difference of < 0.05 from P is considered statistically significant.

## Results

### Identification of gene modules related to BLBC

**Fig. 2:**
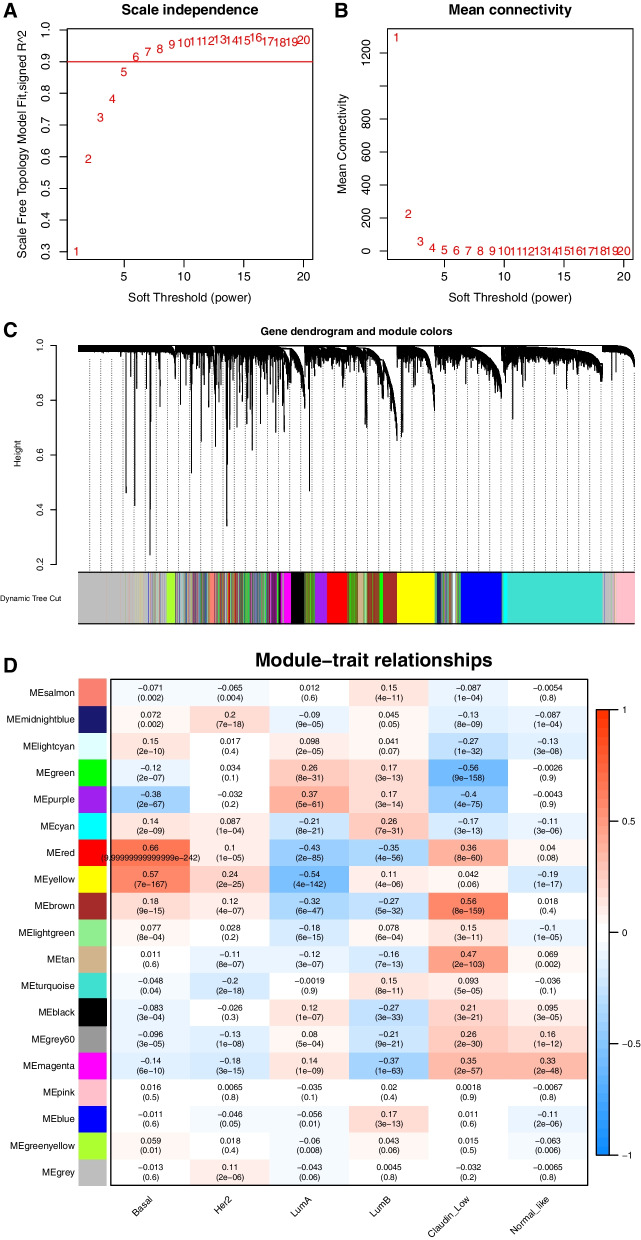
Identification of gene modules related to BLBC by WGCNA. **A** The scale free fit index as a function of the soft-threshold power. **B** The mean connectivity as a function of the soft-threshold power. **C** Clustering dendrogram of genes, with dissimilarity based on the topological overlap, together with assigned module colors. **D** Module-BC subtypes associations. Each row corresponds to a module eigengene, column to a BC subtype. Each cell contains the corresponding correlation and *p*-values. The table is color-coded by correlation based on the color legend

WGCNA was applied to identify gene modules associated with BLBC. The optimal soft threshold was determined with a power of ꞵ = 5 (Fig. 2A and B, the genes were clustered into 19 modules based on the topological overlap (Fig. 2C), and the red module (coefficient = 0.66, *p* < 0.05) showed the highest correlation with BLBC (Fig. 2D), 913 promising candidate genes were identified from the red module.

### Functional enrichment analysis of the candidate genes from ‘red module’

**Fig. 3:**
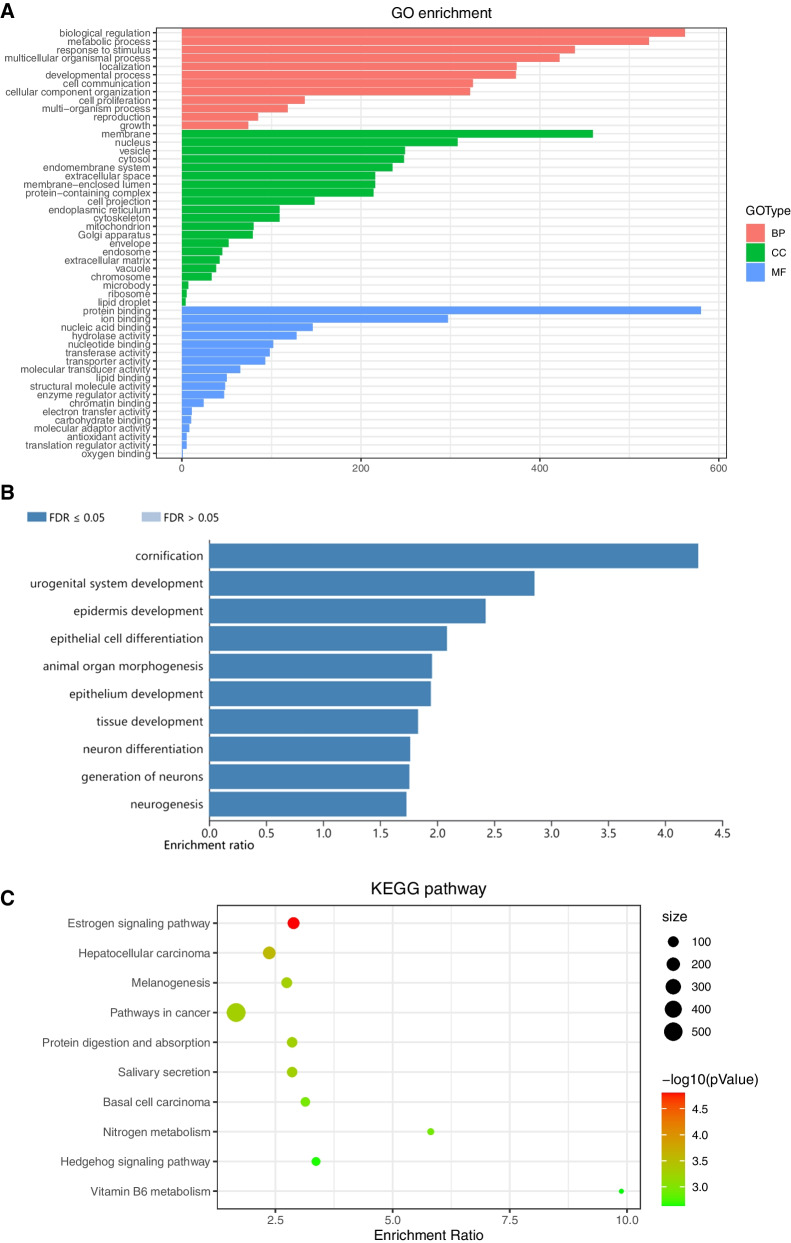
Functional enrichment analysis of the candidate genes related to BLBC in red module. **A** The genes numbers involved in GO terms. **B** Bar graph of the enriched GO terms. **C** Result of KEGG pathway analysis. * The circle size indicates the gene ratio of the candidate genes in the overall pathway and the color indicates the -log 10 (*p* value)

The 913 candidate genes of the red module related with BLBC were involved in multiple GO terms (including 12 BP, 22 CC and 17 MF), such as biological regulation and metabolic process for BP, membrane and nucleus for CC, and protein binding as well as ion binding for MF (Fig. 3A).The TOP10 enriched GO terms for those candidate genes included cornification, epidermis development and epithelial cell differentiation with adjusted FDR (Fig. 3B, *p* < 0.05). What’s more, most results of the KEGG enrichment analysis were mainly enriched in estrogen signaling pathway as well as pathways in cancer (Fig. 3C).

### Identification of DEGs between BLBC and other BC subtypes

**Fig. 4:**
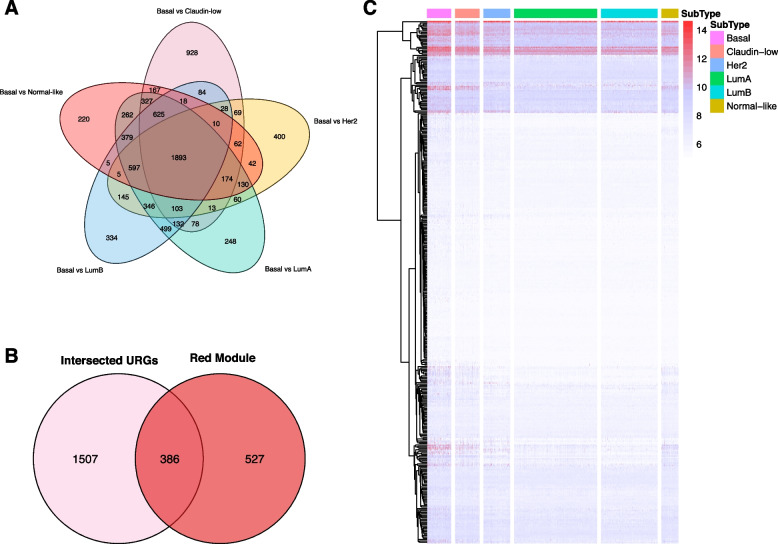
**A** Venn diagram indicating 1,893 common URGs in BLBC compared with other BC subtypes. **B** Venn diagram indicating 386 overlapped candidate genes between the 1893 common URGs and the 913 genes in ‘red module’ identified by WGCNA. **C** Heat map of the 386 candidate genes in each of the BC subtypes

We then intersected the overlapped URGs from each result and identified 1,893 URGs in BLBC compared with other BC subtypes (Fig. 4A). These URGs were further compared with the 913 genes in ‘red module’ identified by WGCNA. Finally, 386 common candidate genes for BLBC were identified (Fig. 4B). The expression heatmap of the 386 candidate genes in each of the BC subtype were shown in Fig. 4C.

### Prognostic value of the 386 overlapped candidate genes associated with BLBC

**Fig. 5:**
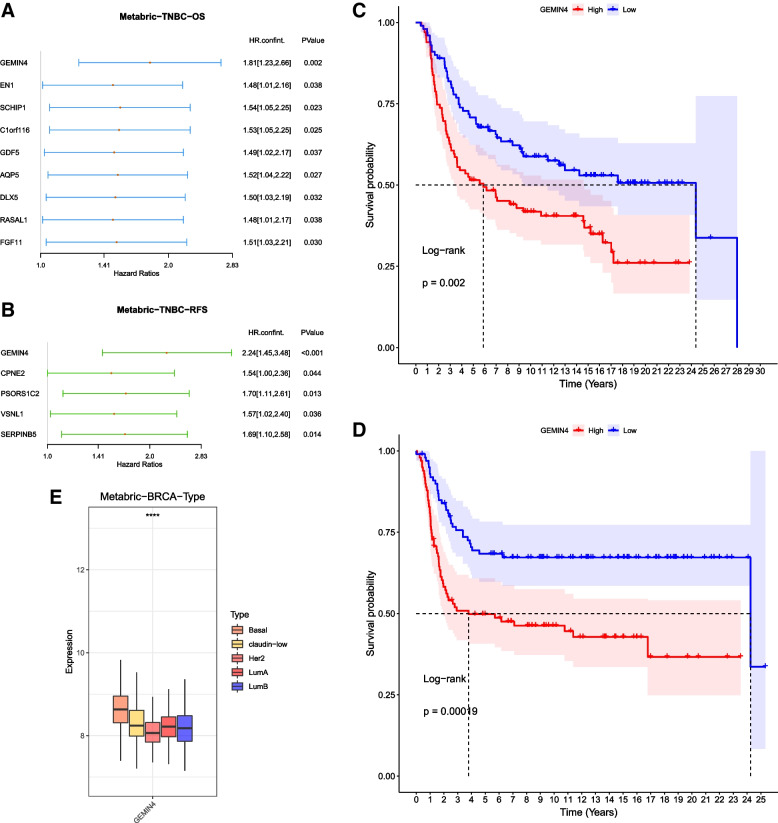
**A-B** Forest plot indicating the highly expressed genes in BLBC with the poor OS and RFS time, respectively. The HR were presented with its 95% confidence interval. **C-D** KM-plots of OS and RFS in the GEMIN4-based groups. Log-rank test was used for data analysis. **E** Box plot indicating the expression level of GEMIN4 in each of the BC subtypes.****p < 0.0001

Based on the median expression value of the 386 candidate genes, patients with BCBL were separated into high-risk as well as low-risk groups. The prognostic value, including OS and RFS, of the 386 candidate genes between the two groups were investigated. In BLBC patients, nine highly expressed genes, including *GEMIN4, EN1, SCHP1, C1orf116, GDF5, AQP5, DLX5, RASAL1* and *FGF11,* were identified to be associated with poor OS time (Fig. 5A). Five highly expressed genes, including *GEMIN4, CPEN2, PSORS1C2, VSNL1* and *SERPINB5*, were associated with poor RFS time (Fig. 5B). As shown in Fig. 5C (OS) and 5D (RFS), the Kaplan–Meier (KM) survival analysis demonstrated high *GEMIN4* (*P* < 0.05) expression level was related to poor prognosis in BLBC patients. The expression level of *GEMIN4* in each BC subtypes were shown in Fig. 5B.

### Biological function of GEMIN4 in BLBC cell lines

**Fig. 6:**
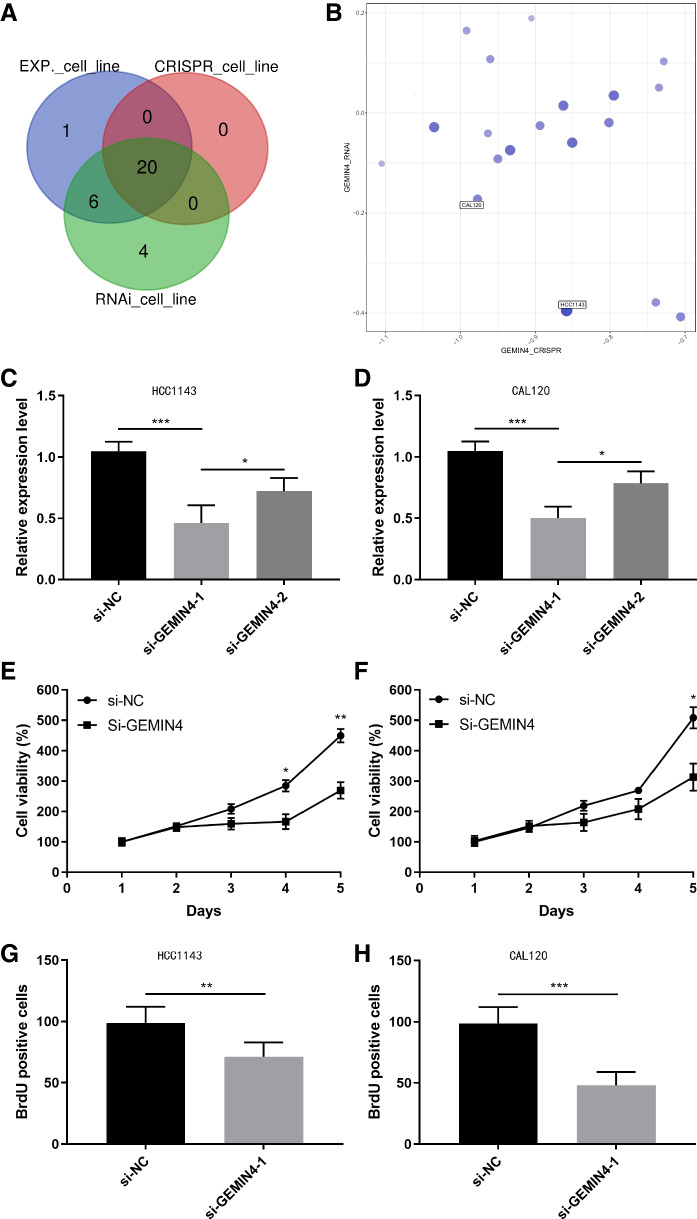
**A** Venn diagram indicating 20 common cell lines of *GEMIN4* dependency. **B** The *GEMIN4* gene dependency scores of CRISPR and RNAi analysis. **C-D** qRT-PCR results of GEMIN4 mRNA levels in si-NC and two GEMIN4 siRNA group in HCC1143 or CAL120 cell lines. **E**–**F** CCK8 assay of si-NC and si-GEMIN4 group in two cell lines. **G**-**H** The BrdU positive cell numbers in different groups. *p < 0.05, **p < 0.01, and ***p < 0.001. Each experiment was repeated at least five times

Based on the average expression of *GEMIN4* in BLBC cell lines as well as the CRISPR, RNAi analysis, 20 *GEMIN4* dependency cell lines were selected (Fig. 6A). As shown in the scatter score plot, the *GEMIN4* gene dependency scores in cell line HCC1143 and CAL120 were negative and lower and the cell survival was inhibited (Fig. 6B). Next, to study the molecular function of GEMIN4 in BC cells, we knock down GEMIN4 expression through siRNA transfection. We designed two siRNA, si-GEMIN4-1 and si-GEMIN4-2, and transfected them into HCC1143 and CAL120 respectively. After 48 h transfected, the qRT-PCR results showed si-GEMIN4-1 group cells presented more decreased GEMIN4 mRNA compared with si-NC (*p* < 0.001, *p* < 0.001) group and si-GEMIN4-2 (*p* < 0.05, *p* < 0.05) both in HCC1143 cells and CAL120 cells (Fig. 6C, D). Thus, we used the si-GEMIN4-1 to knock down GEMIN4 expression in following experiments. Because function analysis data hinted GEMIN4 may associate with DNA metabolic process and ribosome biogenesis, we performed CCK8 assay in GEMIN4 knockdown cells. The findings revealed that the cell viability of si-GEMIN4 group was much lower compared with si-NC group (*p* < 0.05, *p* < 0.001) (Fig. 6E, F). Also, the BrdU staining results presented reduced BrdU positive cells in si-GEMIN4 group compared with si-NC group in HCC1143 and CAL120 cell lines (*p* < 0.01, *p* < 0.001) (Fig. 6G, H). In general, knockdown of GEMIN4 may disturb cell proliferation ability in breast cancer cells.

### Molecular mechanism of GEMIN4 in the progression of BLBC

**Fig. 7:**
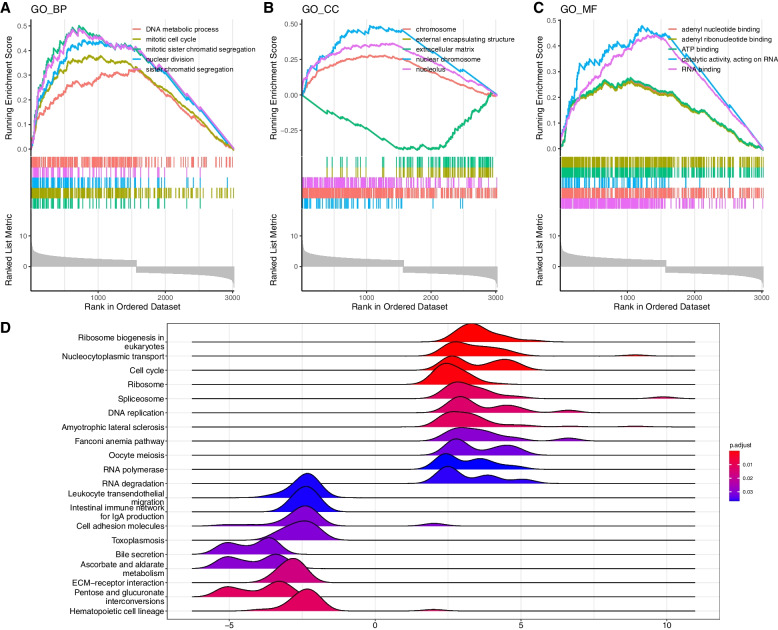
**A-C** GO enrichment analysis of the DEGs grouped by GEMIN4. **D** KEGG enrichment analysis of the DEGs grouped by GEMIN4

In the GO enrichment analysis, GEMIN4 was mainly associated with DNA metabolic process in biological process model, extracellular matrix in cellular component model and adenyl nucleotide binding in molecular function model (Fig. 7A-C). Furthermore, the KEGG pathway enrichment analysis revealed GEMIN4 was related to ribosome biogenesis in eukaryotes (Fig. 7D).

## Discussion

BC has become one of the commonest carcinoma in female worldwide with heavy disease burden. Due to lack of effective therapy strategies, the clinical outcome of BLBC is the poorest among all BC molecular subtypes [13]. Therefore, it is significant to identify the molecules that can be used as diagnostic maker for BLBC at early stage and provide optimized treatment strategies for BLBC patients. In this study, we aimed to explore the treat targets to monitor the progression and prognosis of BLBC.

In this study, we used the WGCNA analysis to find gene modules which related to BLBC disease. WGCNA was a powerful tool to explore co-expression networks and potentially related modules and presented efficiency in cancer-related genetic analysis, the whole genome searching in model species, and functional MRI data analysis [14]. For example, Luo et al. performed the WGCNA analysis in pan-cancer diseases including kidney renal clear cell carcinoma (KIRC), breast invasive carcinoma (BRCA), brain lower grade glioma (LGG), kidney renal papillary cell carcinoma (KIRP), liver hepatocellular carcinoma (LIHC), thyroid carcinoma (THCA), Sarcoma (SARC), as well as lung adenocarcinoma (LUAD) respectively to explore the relationship between coexpression modules and telomerase reverse transcriptase (TERT) expression levels in pan-cancer [15]. Also, through WGCNA analysis of four GEO datasets, Rezaei et al. found the predictive genes ITGAX, CCL14, ADHFE1, and HOXB13 show a close correlation with gastric cancer (GC), and present higher expression levels in GC tissues [16]. In our studies, we used WGCNA analysis to METABRIC datasets and found 19 gene modules.

WGCNA identified multiple candidate genes associated with BLBC and those genes were enriched in cornification, epidermis development, epithelial cell differentiation, estrogen signaling pathway, and pathways in cancer, which is consistent with the pathogenesis of BLBC. For example, the DEGs of triple negative breast cancer were also enriched in epidermis development according to the previous research [17]. In addition, some transcription factors essential for mammary luminal epithelial cell differentiation are also involved in the BLBC and estrogen receptor downstream target gene could inhibit the proliferation as well as migration of BC cells [18, 19].

It is interesting that we identify a candidate gene, gem nuclear organelle associated protein 4 (*GEMIN4*), which was upregulated in BLBC and indicating a poor prognostic for BLBC.*GEMIN4,* located in 17p13.3, belongs to the GEMIN protein family that is participated in a variety of pathological processes. It is portion of a large complex localized to the nucleoli, cytoplasm, also to discrete nuclear bodies named Gemini bodies. According to previous studies, the *GEMIN4* protein was one of the vital molecule in the RNA-induced silencing complex (RISC) that involved in the growth of miRNAs, the recognition and repression of target RNA [20–22]. Therefore, abnormity in the *GEMIN4* might lead to the differential expression of some particular miRNAs that are associated with the malignant tumors. For example, Wan et al. revealed that variations in the *GEMIN4* gene had an underlying impact on DNA repair in the hepatoma cancer cells and leaded to the development of hepatoma carcinoma [23]. Moreover, it has been reported that polymorphisms in *GEMIN4* gene were related to the etiology as well as clinical outcome of multiple cancers like renal carcinoma [24], bladder cancer [25] and ovarian cancer [26].

It has been demonstrated by a recent study that *GEMIN4* rs4968104 were associated with the OS of BC [27]. Here, *GEMIN4* was identified as a new candidate gene upregulated in BLBC and associated with the OS and RFS of BLBC. It has not been reported by previous studies that *GEMIN4* involved in the development of BLBC. We hypothesized that *GEMIN4* may be as a potential biomarker for the prognosis prediction of BLBC. Further validation experiment studies are warranted to develop therapy strategies of BLBC. On this basis, further in vivo experiments are needed to validate the therapeutic mechanism of GEMIN4 as a potential molecular target for BLBC.

## Conclusions

In conclusion, the high expression of *GEMIN4* were related to the poor prognosis of BLBC, and *GEMIN4* might be as a carcinoma-promoting role in BLBC and could be an underlying molecular target for the treatment of BLBC.

## Supplementary Information

**Additional file 1: Supplementary Table 1. **Patients’ information (NA，not available).

## Data Availability

The data of this study were derived from the Breast Cancer (METABRIC, Nature 2012 & Nat Commun 2016) dataset in the cBio Cancer Genomics Portal (cBioportal) database.

## Abbreviations

BLBC: Basal-like breast cancer
BC: Breast cancer
RFS: Relapse free survival
OS: Overall survival
DALY: Disability-adjusted life-year
PR: Progesterone receptor
ER: Estrogen receptor
HER2: Human epidermal growth factor 2 receptor
TNBC: Triple-negative breast cancer
GS: Gene significance
MM: Module membership
MF: Molecular function
CC: Cellular component
BP: Biological process
ORA: Over-representation analysis
HR: Hazard ratio
FBS: Fetal bovine serum
KM: Kaplan–Meier
