# Supplementary material for: GEMIN4, a potential therapeutic targets for patients with basal-like subtype breast cancer
Source: BMC Womens Health. 2023 Jul 28;23:396. doi: 10.1186/s12905-023-02547-1 (PMC10386636; doi:10.1186/s12905-023-02547-1)
Supplement: Supplementary file 1 — Additional file 1: Supplementary Table 1. Patients’ information (NA，not available). [file 12905_2023_2547_MOESM1_ESM.docx]

| **Variables** | | **Counts** |
| --- | --- | --- |
| **Age** | >=50 | 1487 (78.3%) |
|  | <50 | 411 (21.7%) |
| **Gender** | Male | 0 (0%) |
|  | Female | 1898 (100%) |
| **Subtype** | Basal | 199 (10.5%) |
|  | Her2 | 220 (11.6%) |
|  | LumA | 679 (35.8%) |
|  | LumB | 461 (24.3%) |
|  | Normal-like | 140 (7.3%) |
|  | Claudin-low | 199 (10.5%) |
| **Tumor_Stage** | I | 475 (25.0%) |
|  | II | 797 (42.0%) |
|  | III | 115 (6.1%) |
|  | IV | 9 (0.5%) |
|  | NA | 502 (26.4%) |
| **Total of Patients** | | 1898 |

* Table1. Patients’ information (NA，not available)
